# Comparison of diagnostic performance in assessing the rewiring position into a jailed side branch between online 3D reconstruction systems version 1.1 and 1.2 derived from optical frequency domain imaging

**DOI:** 10.1007/s12928-019-00629-2

**Published:** 2019-11-09

**Authors:** Tatsuhiro Fujimura, Takayuki Okamura, Kazuki Furuya, Yosuke Miyazaki, Hitoshi Takenaka, Hiroki Tateishi, Tetsuro Oda, Mamoru Mochizuki, Hitoshi Uchinoumi, Takashi Nishimura, Jutaro Yamada, Masafumi Yano

**Affiliations:** 1 Division of Cardiology, Department of Medicine and Clinical Science, Yamaguchi University Graduate School of Medicine, 1-1-1 Minami-kogushi, Ube, Yamaguchi 755-8505 Japan; 2 Department of Clinical Engineering, Yamaguchi University Hospital, Yamaguchi, Japan

**Keywords:** Coronary bifurcation stenting, Kissing balloon inflation, Agreement comparison, Three-dimensional reconstruction, Optical frequency domain imaging

## Abstract

The three-dimensional reconstruction of optical coherence tomography and optical frequency domain imaging (3D-OCT/OFDI) helps optimize bifurcation percutaneous coronary interventions (PCIs) with side branch (SB) dilatation by identifying the optimal rewiring position. 3D-OCT/OFDI’s diagnostic performance for assessing the rewiring position into a jailed SB is unknown. We retrospectively evaluated the diagnostic performances of a conventional (ver. 1.1) and a new (ver. 1.2) online 3D-OFDI reconstruction system based on an offline 3D reconstruction system’s performance. We analyzed 45 patients’ 52 OFDI pullbacks with main vessel stenting followed by rewiring into a jailed SB for coronary bifurcation lesions. We counted the undetected stent struts in the polygon of confluence as the stent detection performance. We assessed the diagnostic agreement regarding the rewiring position into a jailed SB by the three 3D reconstruction systems. The percentage of undetected struts and the diagnostic agreement of ver.1.2 were significantly better than those of ver.1.1 [5.1 ± 5.1% vs. 30.2 ± 14.2%; *p* < 0.0001, and 94.2% (49/52) vs. 76.9% (40/52); *p* = 0.0120]. The new online 3D-OFDI reconstruction system provides better diagnostic performance than the conventional online system for assessing the rewiring position into a jailed SB.

## Introduction

The three-dimensional reconstruction of optical coherence tomography/optical frequency domain imaging (3D-OCT/OFDI) is useful for identifying the optimal rewiring position (generally a distal cell), resulting in a reduction of incomplete stent apposition (ISA) at bifurcation segments [1, 2] and preserving a good expansion of the side branch ostium area in patients with main vessel (MV) stenting and kissing balloon inflation (KBI) [3], and the utilization of 3D-OCT/OFDI might improve clinical outcomes in terms of the bifurcation percutaneous coronary intervention (PCI). Online 3D-OCT/OFDI has recently become available for use in catheter labs, enabling operators to quickly and easily understand the complexity of the anatomy in bifurcation lesions without the need for dedicated software. During a bifurcation PCI, online 3D-OCT/OFDI is useful for revealing the stent configuration in front of the side branch (SB) ostium and the guidewire re-crossing point. The new online 3D-OFDI reconstruction system (i.e., LUNAWAVE ver. 1.2) was also designed to improve the 3D reconstruction image quality to better assess the relationship between the vessel wall and PCI devices (e.g., the stent, wire, and SB ostium) compared to the conventional online 3D-OFDI reconstruction system (LUNAWAVE ver. 1.1).

In an assessment of the rewiring position by the offline 3D reconstruction system, high feasibility and good inter- and intra-observer agreements have been reported [4]. In contrast, the diagnostic performance of the online 3D-OCT/OFDI reconstruction systems has been unclear. We conducted the present study to investigate both the performance of the ver. 1.1 and ver. 1.2 online systems for detecting stent struts in the polygon of confluence (POC) and the diagnostic agreement for assessing the rewiring position into a jailed side branch after MV stenting, based on the offline 3D reconstruction system derived from OFDI.

## Methods

### Study population

We retrospectively analyzed all consecutive patients who underwent an OFDI-guided bifurcation PCI during the period from July 2013 to September 2017 at Yamaguchi University Hospital. For our present analyses, we extracted patients with an OFDI pullback, which was obtained to assess the guidewire rewiring position at the first attempt after MV stenting crossing over a SB. We excluded cases in which the rewiring position could not be evaluated on a 3D-OFDI image due to the poor image quality.

### OFDI image acquisition

OFDI images were obtained using the LUNAWAVE™ and a FastView™ intravascular imaging catheter (Terumo Corp., Tokyo) after the guidewire’s rewiring into the SB. Automatic pullbacks were performed at 20 mm/s during the contrast injection for blood clearance from the lumen area, using a power injector. The imaging catheter was inserted ≥ 10-mm distal to the MV stent, and the pullback continued until either the imaging catheter reached the guiding catheter, or the automatic pullback was completed.

### The conventional online 3D-OFDI reconstruction system (ver. 1.1), the new online 3D-OFDI reconstruction system (ver. 1.2), and the offline 3D reconstruction system

Three-dimensional reconstructions of OFDI pullbacks were generated by three different systems: (1) the online software installed in the LUNAWAVE system (ver. 1.1), which is the conventional 3D-OFDI reconstruction system; (2) the new online software installed in the LUNAWAVE system (ver. 1.2), which is the new online 3D OFDI reconstruction system; and (3) the offline 3D reconstruction system. Online 3D-OFDI images were reconstructed with both the conventional and the new 3D reconstruction system from the raw OFDI image data. The software on the OFDI console had been already updated to ver. 1.2, and we therefore used the offline workstation, LUNAWAVE Offline viewer (ver. 1.1) (Terumo) for this analysis.

The software program of the offline 3D reconstruction system (ver. 1.1) is identical to that of the online LUNAWAVE system software program. The offline 3D images were reconstructed using the 3D-DICOM viewer INTAGE Realia™ (Cybernet, Tokyo) after stent struts were detected automatically by the dedicated in-house software [5]. The raw data of the OFDI pullbacks were exported to the hard drive and imported to the offline workstation (ver. 1.1) and the offline 3D system.

### Assessment of the incidence of undetected stent struts

For each frame, we counted the number of total stent struts and the number of stent struts that were not detected in the POC when using the ver. 1.1, ver. 1.2, and offline 3D reconstruction systems. Each incidence of undetected stent struts to total stent struts (% undetected struts) were calculated and compared.

### Assessment of the rewiring position

We retrospectively analyzed the overhanging stent configuration and the rewiring point using the three 3D reconstruction imaging systems. Both the overhanging stent configuration and the rewiring point were assessed on a ‘cut-away’ view and/or a carpet view [6, 7]. We divided the overhanging stent configurations into two types (the link-free carina type and the link-connecting-to-the-carina type) based on the presence/absence of a longitudinal stent link at the carina. In the link-free carina type, there is no longitudinal stent link between crowns at the carina. In the link-connecting-to-the-carina type, there is a longitudinal link connecting to the carina.

**Fig. 1:**
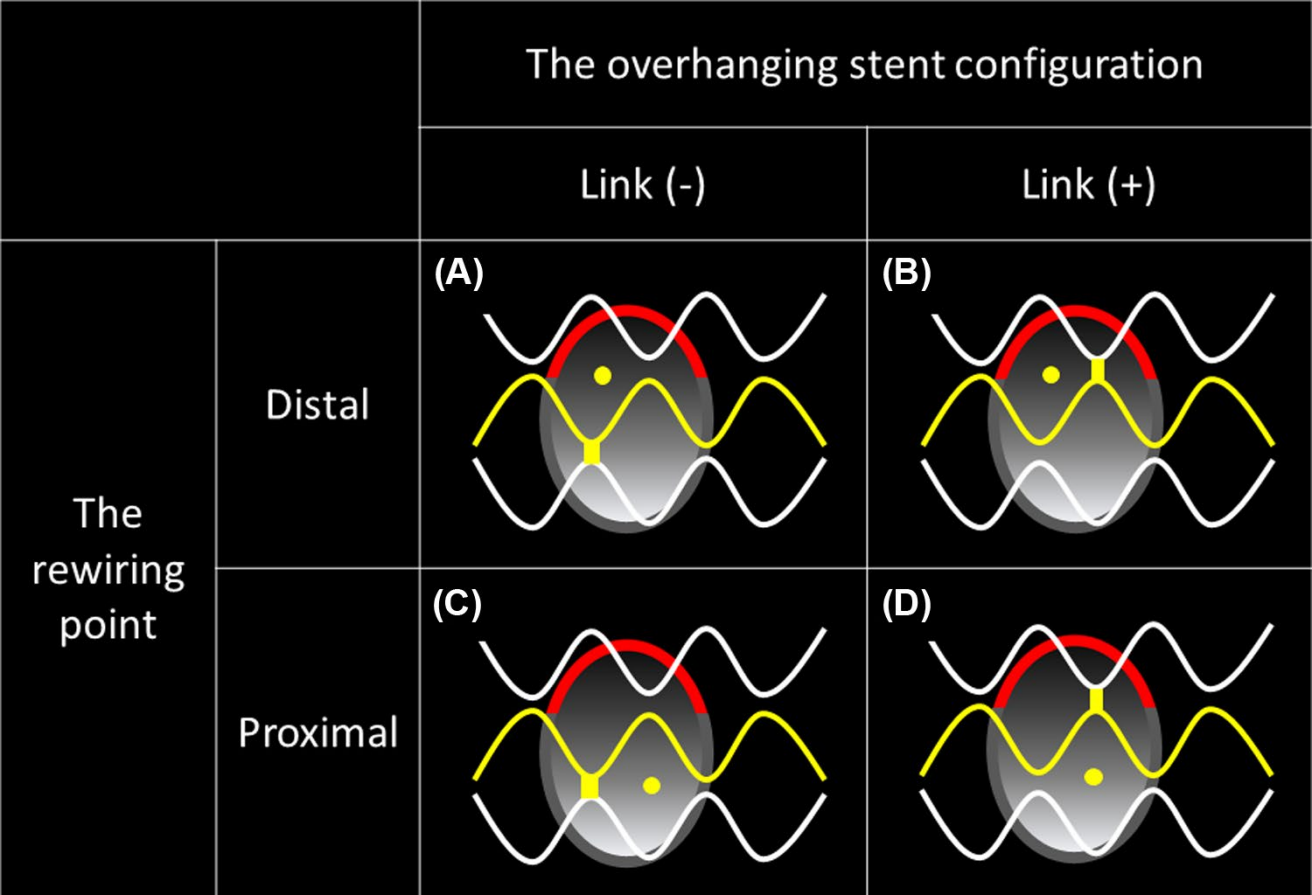
The assessment of the rewiring position in front of the side branch ostium. Yellow dots: rewiring points. Red lines: bifurcation carinas. Yellow stent struts: stent struts with at least one distal top of the stent hoop located in front of the side branch ostium. **a** Distal cell rewiring with the link-free carina type. **b** Distal cell rewiring with the link-connecting-to-the-carina type. **c** Proximal rewiring with the link-free carina type. **d** Proximal rewiring with link-connecting-to-the-carina type

We divided the rewiring points into distal and proximal rewiring points. The distal cell was defined as a stent strut having at least one distal top of the stent hoop located in front of the SB ostium to the distal side [1]. As shown in Fig. 1, we classified the 52 OFDI pullback images into four categories according to the overhanging stent configuration and rewiring point: (A) distal cell rewiring with the link-free carina type, (B) distal cell rewiring with the link-connecting-to-the-carina type, (C) proximal rewiring with the link-free carina type, and (D) proximal rewiring with the link-connecting-to-the-carina type. We evaluated the agreement of the four categories assessed by the two online 3D-OFDI reconstruction systems (ver. 1.1 and ver. 1.2) against that assessed by the offline 3D system, and then we compared the agreements between ver. 1.1 and the offline 3D reconstruction system and between ver. 1.2. and the offline 3D reconstruction system.

### Inter- and intra-observer reproducibility assessment

We evaluated the inter- and intra-observer reproducibility of the assessments of the overhanging stent configuration and the rewiring point in front of the SB ostium. Inter-observer reproducibility was assessed in all cases by a dedicated interpreter (T.F.) and an independent second interpreter (K.F.). Intra-observer reproducibility was assessed in all cases at ≥ 4 weeks after the initial evaluation.

### Statistical analysis

Continuous variables are presented as the mean value and standard deviation (SD) if normally distributed, or as medians and interquartile ranges (IQRs) if not normally distributed. The data were assessed using Student’s *t* test when the data were normally distributed and the nonparametric Kruskal–Wallis rank sum test when the data were not normally distributed. Categorical variables were compared between groups by *χ*^2^ test or Fisher’s exact test. *P* values < 0.05 were considered significant. Inter-observer and intra-observer analyses were performed using Cohen’s kappa coefficient. The interpretation of the kappa coefficient values was as follows: a kappa value of 0.81–1.00 indicates almost perfect agreement; a value of 0.61–0.80 indicates substantial agreement, and a value of 0.41–0.60 indicates moderate agreement [8]. All statistics were calculated using JMP ver. 13 software (SAS Institute, Cary, NC, USA).

## Results

### Inclusion and exclusion

Intracoronary imaging devices were used in 995 PCIs (88%) out of 1,127 PCIs performed in our institution from 2013 to 2017. OFDI was used in 193 PCIs (17.1%). There were 89 bifurcation lesions. Of these, the guidewire rewiring position was checked by OFDI after stent implantation for a main vessel in 48 cases. Fifty-six OFDI pullbacks in 48 PCIs (single stenting *n* = 40, culotte stenting *n* = 8) were acquired immediately after rewiring. Four pullbacks in three cases were excluded due to the poor image quality in all three 3D systems (i.e., inadequate blood clearance, artifacts of guidewire shadow, and cardiac motion hindering the visualization of the SB ostium). A final total of 52 OFDI pullbacks in 45 PCIs were investigated in the present study. Thus, the feasibility of 3D reconstruction was 92.9% (52/56). Seven pullbacks (seven patients) were obtained after the second stent deployment and rewiring in culotte stenting.

### Patient, lesion, and procedure characteristics

The patient and lesion characteristics and the procedure details of the pullbacks are summarized in Table 1. Thirteen patients with acute coronary syndrome (ACS) were included (28.9%). Left main bifurcations were present in 37 of the 45 patients (82.2%). The incidence of true bifurcation lesions was 37.8% (17 of the 45 patients). The proximal optimization technique before rewiring was performed in 40 of the 52 pullbacks (76.9%).

**Table 1:** Patient and lesion characteristics

Patients, *n*	45
Age, yrs; median (IQRs)	70 [64–81]
Male, *n* (%)	34 (75.5)
Primary disease, *n* (%):	
ACS	13 (28.8)
SAP	13 (28.8)
SI	17 (37.7)
OMI	2 (4.4)
Risk factor, *n* (%):	
Hypertension	33 (73.3)
Dyslipidemia	33 (73.3)
Diabetes mellitus	24 (53.3)
Smoking	31 (68.8)
Target bifurcation, *n* (%):	
LM	37 (82.2)
LAD-D	4 (8.8)
LCX-OM	3 (6.6)
RCA PD-PL	1 (2.2)
Medina classification, *n* (%):	
(1.1.1)	12 (26.6)
(1.1.0)	3 (6.6)
(1.0.1)	1 (2.2)
(1.0.0)	2 (4.4)
(0.1.1)	4 (8.8)
(0.1.0)	23 (51.1)
(0.0.1)	0 (0.0)
True bifurcation, *n* (%)	17 (37.8)
OFDI acquisition	
Flushing material; contrast	45 (100)
Pullback speed: 20 mm/s	45 (100)
Two-stent technique, *n* (%)	7 (15.5)
Pre-dilatation for SB before MV stenting	
Sequential dilatation, *n* (%)	7 (15.6)
Kissing balloon dilatation, *n* (%)	5 (11.1)
No SB pre-dilatation, *n* (%)	33 (73.3)
Analyzed pullbacks, *n*	52
Main vessel	
LM-LAD (SB: LCx)	32 (61.5)
LM-LCx (SB: LAD)	10 (19.2)
LAD	5 (9.6)
LCx	4 (7.6)
RCA	1 (1.9)
Stent type of MV, *n* (%):	
BMX-J™	23 (44.2)
Resolute™	11 (21.1)
Ultimaster™	13 (25.0)
Synergy/Promus™	5 (9.6)
Stent size, mm, median [IQRs]	3.5 [2.75–3.5]
Stent length, mm, median [IQRs]	18 [16.5–24]
POT Before rewiring, *n* (%)	40 (76.9)
Usage of double lumen catheter, *n* (%)	43 (82.7)

*ACS* acute coronary syndrome, *D* diagonal coronary artery, *LAD* left anterior descending coronary artery, *LCX* left circumflex coronary artery, *LM* left main coronary artery, *MV* main vessel, *OFDI* optical frequency domain imaging, *OM* obtuse marginal, *OMI* old myocardial infarction, *PD* posterior descending, *PL* posterolateral, *POT* proximal optimization technique, *RCA* right coronary artery, *SAP* Stable angina pectoris, *SB* side branch, *SD* standard deviation, *SI* silent ischemia

### Comparison of the incidence of undetected stent struts

**Fig. 2:**
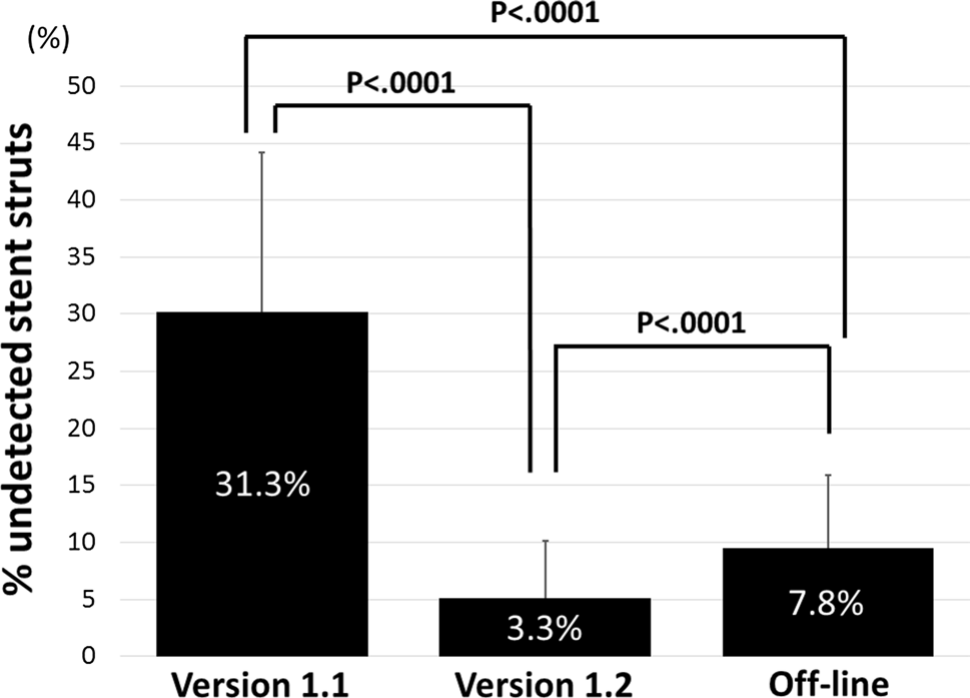
The assessment of the incidence of undetected stent struts in the polygon of confluence for each system

The incidence of undetected struts in each system is illustrated in Fig. 2. Among the three 3D reconstruction imaging systems, the ver. 1.2 system provided the lowest incidence of undetected stent struts [31.3% (19, 38.4) in ver. 1.1 vs. 3.3% (2.0, 6.7) in ver. 1.2 vs. 7.8% (4.1, 13.4) in offline, *p* < 0.0001].

### Comparison of agreement with the offline 3D system

**Fig. 3:**
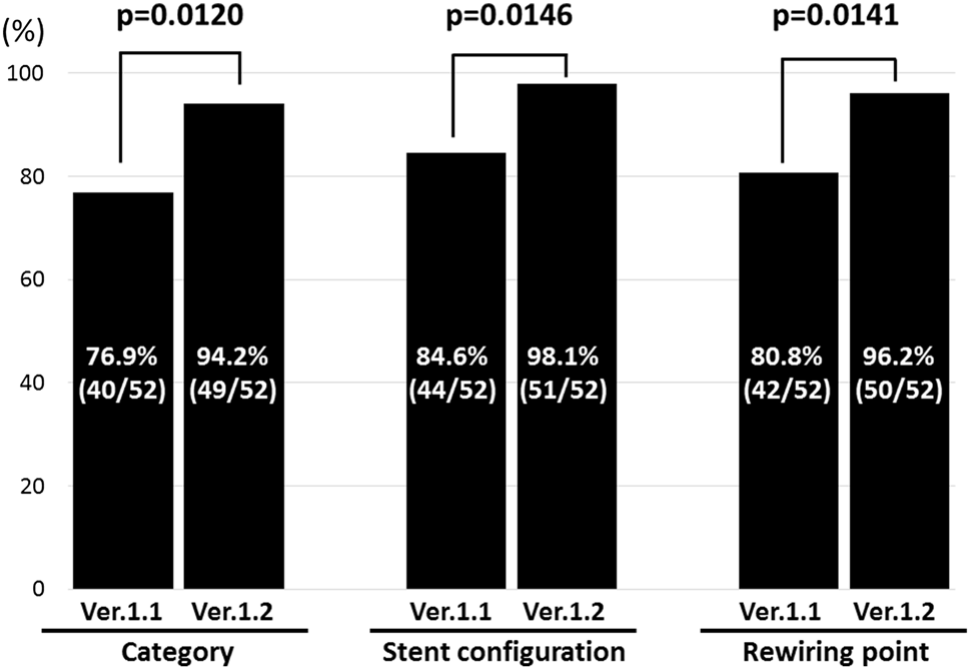
Comparison of agreement between ver. 1.1 (the conventional online 3D-OFDI) and ver. 1.2 (the new online 3D-OFDI) for the four categories of the overhanging stent configuration and rewiring point together, the stent configuration, and the rewiring point

The diagnostic agreement of the categories assessed by the ver. 1.2 system was significantly better than that assessed by the ver. 1.1 system: 94.2% (49/52) vs. 76.9% (40/52), respectively, *p* = 0.0120. Regarding the overhanging stent configuration (i.e., link-free carina type or link-connecting-to-the-carina type), the diagnostic agreement was 84.6% (44/52) for the ver. 1.1 system, which was significantly less than that for the ver. 1.2 system at 98.1% (51/52) (*p* = 0.0146). The diagnostic agreement regarding the rewiring points (i.e., distal cell rewiring or proximal cell rewiring) was significantly increased from 80.8% (42/52) in ver. 1.1 to 96.2% (50/52) in ver. 1.2 (*p* = 0.0141) (Fig. 3).

**Fig. 4:**
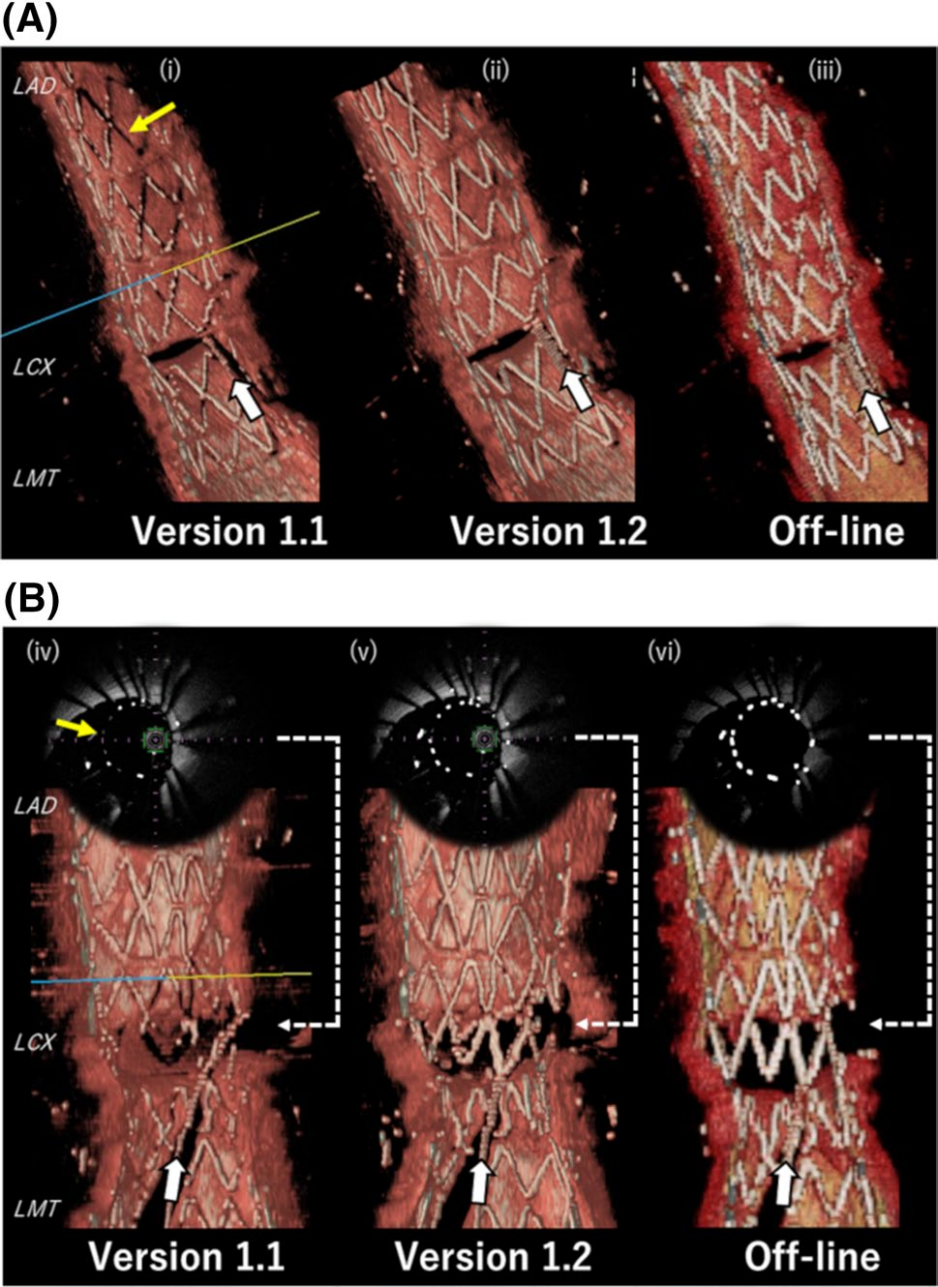
Representative images from each of the three 3D reconstruction systems. **a** Representative pullback 1, a case of agreement by all three systems. White arrows: the guidewire of rewiring into the side branch. Yellow arrows: the undetected stent struts. **b** Representative pullback 2, a case of disagreement by ver. 1.1. (iv) and (v) are the online cross-sectional OFDI image with the stent struts highlighted. (vi) is the cross-sectional OFDI imaging using in-home software with the stent strut highlighted. The stent struts were not detected sufficiently by ver. 1.1 (iv), whereas they were detected almost perfectly by ver. 1.2 (v) and the offline 3D system (vi)

Figure 4a shows a representative case of agreement by all three systems. Although the stent detection by the ver. 1.1 system was not sufficient, the overhanging stent configuration and rewiring point were assessable by all three systems. Figure 4b shows a representative case of disagreement. The diagnosis of both the overhanging stent configuration and the rewiring point assessed by the ver. 1.1 system were not in agreement with those assessed by the offline 3D imaging system due to the invisible struts at the bifurcation. However, compared to ver. 1.1, the ver. 1.2 system was able to reconstruct the stent struts clearly, enabling the diagnosis of the stent configuration and rewiring point.

### Reproducibility assessment of the rewiring position

The inter-observer kappa coefficients among the ver. 1.1, ver. 1.2, and offline 3D reconstruction systems were 0.67, 0.85, and 0.82, respectively. The intra-observer kappa coefficients among the ver. 1.1, ver. 1.2, and offline 3D reconstruction systems were 0.82, 0.94, and 0.88, respectively.

## Discussion

The main findings of this study were as follows: (1) the percentage of stent struts not detected by the ver. 1.2 reconstruction system was significantly the lowest among the three 3D reconstruction systems, (2) the diagnostic agreement between ver. 1.2 and the offline system was better than that between ver. 1.1 and the offline system, (3) the inter- and intra-observer reproducibility assessment showed almost perfect agreement in the assessment with ver. 1.2, whereas ver. 1.1 was in only substantial agreement.

This study is a first report delineating the stent detection performance in each of three 3D reconstruction systems and the diagnostic agreements of the 3D-OFDI reconstruction systems in assessing the rewiring position into a jailed side branch after main vessel stenting in a comparison with the offline 3D reconstruction system. To visualize stent struts on 3D-reconstructed images of OCT/OFDI, it is necessary to detect and emphasize the stent struts in each frame [9, 10]. In the current online 3D-OCT/OFDI system, the detection and emphasis of stent struts are processed internally within the online software. Therefore, the image quality of 3D-OCT/OFDI depends on its performance at both detecting and emphasizing the stent strut. Although the stent struts attached to the coronary vessel wall were not detected accordingly, those stent struts can be recognized as the shadow on the vessel wall, enabling operators to observe the stent struts. However, if stent struts in front of the SB ostium are insufficiently detected, the stent struts cannot be identified. Thus, high system performance for detecting stent struts is needed. Our present analyses demonstrated that ver. 1.2 has the highest stent detection performance and better diagnostic agreement compared to ver. 1.1.

These findings suggested that the diagnostic accuracy of the ver. 1.2 system would be higher than that of ver. 1.1 regarding stent configuration in front of the SB and the rewiring point, which are highly needed in bifurcation PCIs. As shown in Fig. 4b, the detection of stent struts by ver. 1.2 was better than that by ver. 1.1. This demonstrates that ver. 1.2 improved the performance to detect and/or emphasize stent struts, resulting in better 3D images. The diagnostic accuracy has thus been improved in ver. 1.2.

Regarding KBI, although the routine KBI has no clinical advantage for bifurcation lesions, further intervention for a side branch is required when a suboptimal result in the side branch ostium such as slow/no flow and a low fractional flow reserve value is clearly expected and recognized [11, 12]. The optimal rewiring technique using 3D-OCT/OFDI is useful for optimal side branch dilatation when it is needed. Although the distal re-crossing is generally recommended [13–15], it was recently reported that the frequency of distal rewiring was 69.9% at the first attempt under angiography [4]. It was also reported that the achievement rate of distal rewiring using offline 3D-OCT was significantly higher than that using 2D-OCT [16]. The use of 3D-OCT/OFDI could therefore be important in optimizing bifurcation PCIs.

A previous study demonstrated that in an assessment of the rewiring position by the offline 3D reconstruction system, the feasibility was 89.8% and the inter- and intra-observer agreements were 0.90 and 0.86, respectively [4]. In the present study, the inter- and intra-observer agreements in the diagnostic performance of the rewiring position by the new version of the online 3D-OFDI system (ver. 1.2) were improved compared to the previous version (ver. 1.1). A repeat acquisition of re-crossing for an unclear 3D image may potentially increase the contrast volume, the radiation time, and the operation time. A misleading image of a rewiring position may potentially inflict unexpected stent deformation at the bifurcation area [17]. Therefore, this improvement would be useful for bifurcation PCIs.

In the latest meta-analysis of five randomized studies of KBI, although the KBI strategy reduced the incidence of side branch restenosis, there was no significant difference in clinical outcomes such as cardiac death, myocardial infarction, stent thrombosis, target lesion revascularization (TLR), and target vessel revascularization (TVR) between the KBI strategy and a no-KBI strategy for coronary bifurcation lesions in the one-stent approach [18]. However, the optimal rewiring afforded by a 3D reconstruction system with high diagnostic performance might be able to change the results of bifurcation PCIs. The optimal rewiring position into a jailed side branch might affect the clinical outcomes, because it can reduce ISA at the bifurcation segment that is associated with thrombotic events [19–22] and preserve a good expansion of the side branch ostium area after a procedure [1, 3]. Prospective and randomized clinical trials are necessary to further investigate the usefulness of 3D-OCT/OFDI in bifurcation PCIs.

## Limitations

Several limitations of this study should be noted. First, there was a potential risk of selection bias because this was a retrospective study at a single center. Second, we could not compare other factors (i.e., the clinical status, lesion type, stent type and proximal optimization technique; POT or not) that could affect the diagnostic performance due to the small number of patients (*n* = 45). Third, the diagnostic performance of the offline 3D reconstruction software was not validated, as there was no way of knowing the “true” rewiring position. However, we used the offline 3D reconstruction software as a base for comparing the two versions (1.1 and 1.2) of the online reconstruction software, because it has been used and reported as a modality to evaluate rewiring positions in the past. The impact of the different software assessments on clinical outcomes after PCIs was not evaluated. A larger number of cases at multiple centers should be examined and prospective studies should be conducted to overcome these study limitations.

## Conclusion

Compared to the conventional online 3D-OFDI reconstruction system, the new online 3D-OFDI reconstruction system provides better diagnostic performance for assessing the rewiring position into a jailed SB.

## Abbreviations

3D-OFDI: Three-dimensional reconstruction of optical frequency domain imaging
ISA: Incomplete stent apposition
KBI: Kissing balloon inflation
MV: Main vessel
OCT: Optical coherence tomography
PCI: Percutaneous coronary intervention
SB: Side branch
